# Influence of Oral Microbiota on the Presence of IgA Anti-Citrullinated Protein Antibodies in Gingival Crevicular Fluid

**DOI:** 10.3389/froh.2022.904711

**Published:** 2022-06-16

**Authors:** Menke J. de Smit, Poerwati Soetji Rahajoe, Elisabeth Raveling-Eelsing, Paola Lisotto, Hermie J. M. Harmsen, Nyoman Kertia, Arjan Vissink, Johanna Westra

**Affiliations:** ^1^Department of Dentistry, University Medical Center Groningen, University of Groningen, Groningen, Netherlands; ^2^Department of Oral and Maxillofacial Surgery, University Medical Center Groningen, University of Groningen, Groningen, Netherlands; ^3^Department of Oral Surgery, Dr. Sardjito General Hospital, Yogyakarta, Indonesia; ^4^Department of Rheumatology and Clinical Immunology, University Medical Center Groningen, University of Groningen, Groningen, Netherlands; ^5^Department of Medical Microbiology, University Medical Center Groningen, University of Groningen, Groningen, Netherlands; ^6^Department of Rheumatology, Dr. Sardjito General Hospital, Yogyakarta, Indonesia

**Keywords:** rheumatoid arthritis, anti-citrullinated protein antibodies (ACPA), periodontitis, mucosal inflammation, microbiome and dysbiosis

## Abstract

**Introduction:**

The relation between rheumatoid arthritis (RA) and periodontitis (PD) has been investigated ever since the discovery of the citrullinating enzyme peptidyl arginine deaminase presents in the oral bacterium *Porphyromonas gingivalis*. Recently, we demonstrated the presence of RA autoantibodies, especially of IgA anti-citrullinated protein antibody (ACPA), in gingival crevicular fluid (GCF) of Indonesian patients with and without RA or PD which might indicate the local formation of RA antibodies in the periodontium.

**Aim:**

The purpose of this study was to assess whether the subgingival microbiome is related to the presence of IgA ACPA in the GCF of healthy individuals with or without PD.

**Patients and Methods:**

Healthy individuals with a known periodontal status and high IgA ACPA (>0.1 U/ml) in GCF (*n* = 27) were selected and matched for age, gender, periodontal status, and smoking status with 27 healthy individuals without IgA ACPA in their GCF. Taxonomic profiling of the subgingival microbiome was based on bacterial 16S rRNA gene sequencing. Downstream analyses were performed to assess compositional differences between healthy subjects with or without IgA ACPA in GCF and with or without PD.

**Results:**

Between groups with or without PD, or with or without IgA ACPA in GCF, no differences in alpha diversity were seen. Beta diversity was different between groups with or without PD (*p* < 0.0001), and a trend was seen in subjects with PD between subjects with or without IgA ACPA in GCF (*p* = 0.084). Linear discriminant analysis effect size (LEfSe) revealed no significant differences in the total population between subjects with IgA ACPA compared to subjects without IgA ACPA in GCF. Although *Porphyromonas* was not identified by LEfSe, its relative abundance was significantly higher in healthy individuals with high IgA ACPA in GCF compared to individuals without IgA ACPA in GCF (*p* = 0.0363). Zooming in on the subgroup with PD, LEfSe revealed that species *Neisseriaceae, Tannerella*, and *Haemophilus* were more abundant in the subjects with IgA ACPA in GCF compared to subjects without IgA ACPA in GCF.

**Conclusion:**

Periodontitis and certain taxa, including *Porphyromonas*, seem to be associated with the local presence of ACPA in the periodontium.

## Introduction

The association between rheumatoid arthritis (RA) and periodontitis (PD) has been investigated for decades and was intensified since Rosenstein in 2004 hypothesized that the oral bacterium *Porphyromonas gingivalis* could have an active role in the onset and progression of RA by the citrullination process mediated by its citrullinating enzyme peptidyl arginine deaminase (PAD) [1]. Antibodies against citrullinated proteins (ACPAs) are a hallmark of RA and are associated with worse disease outcomes [2]. Seropositivity for ACPA and rheumatoid factor (RF) can be detected years before the first signs of joint involvement [3]. The etiology of the immune dysregulation and autoimmunity in RA is therefore presumed to be initiated outside the joint, for example, at inflamed mucosal surfaces, specifically at the mucosal surfaces of the lungs and oral cavity (i.e., the periodontium), in combination with genetic predisposition and environmental factors such as smoking [4]. The fact that RA and PD share genetic and environmental risk factors (smoking, infection) and that in both diseases, inflammation and bone and soft tissue degradation play an important role contributed to increase the number of papers that addresses this relationship. These common features, however, make it very difficult to answer questions about a potential causative link between the two diseases [5].

The presumed bidirectional association between RA and PD has been summarized in the systematic review and meta-analysis by Hussain et al. [6]. There is consistent evidence that PD is associated with higher RA disease activity, whereas RA disease activity does not have an effect on severity of PD [6]. The bidirectional association between RA and PD can also be found in the effect of treatment of one condition on the other [7, 8]. A review of literature concerning cytokines in the local inflammatory exudate of the periodontium (gingival crevicular fluid, GCF) of patients with RA revealed also a bidirectional relationship between RA and PD, probably caused by a non-specific inflammatory burden [9].

Thus, the question whether PD or specific periodontal pathogens are essential in the onset of RA still remains. Dysbiosis of the microbiota in the gut and/or oral cavity has been intrinsically implicated in the development of several immune disorders, including RA [10]. A particular role for the periodontal pathogens *Porphyromonas gingivalis* and *Aggregatibacter actinomycetemcomitans* has been suggested, as these bacteria could initiate the formation of citrullinated proteins and subsequently the formation of ACPA [11, 12].

Contribution of the oral microbiota to the etiopathogenesis of RA was recently discussed by Berthelot et al. [13]. They stated that a growing body of evidence supports that oral bacteria, such as *Porphyromonas gingivalis*, contribute to the pathogenesis of RA, although the genetic background of patients may also play a role [13]. Studies relying on the high-throughput sequencing 16S ribosomal DNA gene ampliconspointed to *Prevotella* as a periodontal taxa of interest among the oral microbiota of patients with RA [14, 15].

As mentioned above, ACPA are indicative of the development of RA, and ACPA might be initiated at mucosal sites. RA-related autoantibodies have been detected at several mucosal sites in populations at risk for developing RA, even in the absence of serum autoantibodies. As not all RA at risk individuals with local ACPA develop RA, Holers et al. discussed in their “mucosal origins hypothesis” that mucosal (IgA) ACPA could have a biologically relevant protective role in normal homeostasis, but that chronic mucosal inflammation and dysbiosis may lead to systemic ACPA expression through multiple potential processes [16].

Thereupon, our group investigated RA-associated autoantibodies in the GCF and serum of patients with RA and healthy controls with or without PD [17] and found that, whereas all autoantibodies including IgA ACPA in GCF of patients with RA were highly correlated with autoantibodies in serum, IgA-ACPA in the GCF of HC was not correlated with systemic levels, but related to inflammation and smoking. These findings led to the aim of this study: to determine whether the presence of IgA ACPA in GCF of healthy subjects is associated with subgingival presence of certain periodontal pathogens.

## Patients and Methods

### Study Subjects

The included subjects were derived from a previously described cohort of healthy individuals. They were selected for an observational study of our group, in which the STROBE guidelines for human observational studies were followed [17]. For this study, the 27 healthy individuals with known periodontal status, who had a high IgA ACPA (>0.1 U/ml) in their gingival crevicular fluid (GCF), were selected. IgA ACPA was measured by modifications to the anti-CCP2 kit (Euro Diagnostica) and described in detail previously [18]. A total of 27 healthy control subjects, matched according to age, gender, smoking, and periodontal status, were selected among the 124 healthy individuals with no or low IgA ACPA in their GCF (<0.1 U/ml). The control subjects were consecutively selected from subjects with an appointment for a first consultation for a third molar assessment or tooth extraction at the Oral and Maxillofacial Surgery Clinic, Dr. Sardjito General Hospital, or the Dental Hospital, Gadjah Mada University in Yogyakarta, Indonesia [17]. All subjects were of Indonesian origin. Exclusion criteria were as follows: under 18 years of age, edentulism, systemic disease (rheumatoid arthritis, diabetes, cardiovascular disease with anticoagulant medication), the presence of non-oral infection, the presence of oral infection other than periodontitis, antibiotic use <3 months prior to the study, the presence of malignancy, and pregnancy including a 6-month postpartum period, as well as breastfeeding.

The study was reviewed and approved by the Medical and Health Research Ethics Committee of the Medical Faculty of Gadjah Mada University, Yogyakarta, Indonesia, according to the Declaration of Helsinki 2008 (Ref: KE/FK/430/EC). All patients were informed verbally and by letter, and the participants provided written informed consent.

### Periodontal Examination and Subgingival Microbial Sampling

Full mouth periodontal measures (pocket probing depth, bleeding on probing, and periodontal attachment loss) were used to calculate the periodontal inflamed surface area (PISA) [19, 20]. Periodontal examination and subgingival microbial sampling were done in the same visit and were supervised by the same examiner (PSR) who was calibrated by a periodontist (MJdS) that was trained in performing PISA and subgingival sampling. Subgingival microbial samples were taken from the deepest bleeding site per quadrant, based on pocket probing depth measurements. If there were no bleeding periodontal, pockets, the mesial site of the first molar, or in the absence of the first molar, the mesial site from the adjacent anterior tooth in the dental arch was selected.

### DNA Extraction and 16S rRNA Gene Sequencing

Subgingival microbial sampling, DNA isolation, purification, quantification, and preparation for Illumina MiSeq were described by Rahajoe et al. [20]. In short, subgingival microbial samples with sterile paper points were based on pocket probing depth measurements and bleeding on probing. Paper points were pooled per patient, and DNA was isolated with a DNA isolation kit (DNeasy Blood & Tissue Kit, Qiagen) after mechanical lysis with zirconia beads (BioSpec Products Inc., Bartlesville, OK, USA). The purity and concentration of the DNA were measured with a NanoDrop 1000 spectrophotometer (Thermo Scientific, Waltham, MA, USA) and Illumina MiSeq preparation and sequencing and were performed as described by Heida et al. [21]. The V3-V4 region of bacterial 16S rRNA genes was amplified by PCR with modified 341F and 806R primers containing a 6-nucleotide index sequence on the 806R primer [22].

### Amplicon Analysis

For taxonomic profiling based of amplicon data, the CosmosID 16S data analysis pipeline (CosmosID Metagenomics Cloud, app.cosmosid.com, CosmosID Inc., www.cosmosid.com) was used which starts with preprocessing of the raw reads from the paired-end FASTQ files through read-trimming to remove adapters as well as reads and bases of low quality. The forward and reverse overlapping pairs were joined together, and the unjoined R1 and R2 reads were added to the end of the file. The file was converted to a FASTA format used as input for OTU picking. Taxonomic assignment was achieved by aligning OTUs against the CosmosID curated 16S database using a closed-reference OTU picker and 97% sequence similarity through the QIIME framework. The results are presented with the taxonomic names, OTU IDs, frequency, and relative abundance (%), which is a normalized metric taking into consideration genome size and number of reads.

### Downstream Analysis

For taxonomic comparative statistical analyses, relative abundance taxonomic matrices and counts per million reads (CPM) functional annotation tables were used as input. Chao's and Shannon's alpha diversity metrics were calculated in R using the R package Vegan (version 2.5-6, https://CRAN.R-project.org/package=vegan). Wilcoxon rank-sum tests were performed between groups using the R package ggsignif (version 0.6.0., https://CRAN.R-project.org/package=ggsignif). Beta diversity (Bray–Curtis) was calculated in R using the R package Vegan with the functions vegdist, and principal coordinate analysis (PCoA) tables were generated using ape's function pcoa (https://cran.r-project.org/package=ape). Permutational multivariate analyses of variance (PERMANOVA) tests for each distance matrix were generated using vegan's function adonis2. Plots were visualized using the R package ggpubr (https://CRAN.R-project.org/package=ggpubr). Holm–Bonferroni correction for multiple comparison was applied.

Differential abundance between cohorts was further assessed by linear discriminant analysis (LDA) effect size (LEfSe). LEfSe is calculated with a Kruskal–Wallis alpha value of 0.05, a Wilcoxon alpha value of 0.05, and a logarithmic LDA score threshold of 2.0. LEfSe figures were generated using the LEfSe tool from the Huttenhower laboratory implemented in the R package lefser (version 1.0.0. https://github.com/waldronlab/lefser).

### Comparative Analysis of Metadata

Group comparisons of metadata were performed using GraphPad Prism (version 8 for Windows; GraphPad Software Inc., La Jolla, CA, USA). Continuous variables were compared with a Mann–Whiney *U*-test when data were not normally distributed (based on Q-Q plots). Normally distributed variables were compared with an unpaired *t*-test with Welch's correction. The significance level of alpha was 0.05.

## Results

### Metadata of Study Subjects

Subjects were divided into IgA ACPA high (IgA ACPA in GCF > 0.1 U/ml) (*n* = 27) and IgA ACPA low groups (IgA ACPA in GCF <0.1 U/ml) (*n* = 27). Metadata of the study subjects are listed in Table 1. Except for IgA ACPA in GCF, groups were similar for all metadata. All subjects were seronegative for RA autoantibodies (cutoff for seropositivity: IgA ACPA > 1.2 U/ml, IgG ACPA > 10 U/ml, IgA RF > 25 IU/ml, and IgM RF > 5 IU/ml). A subdivision was made in group 1 [subjects without periodontal disease (PD), i.e., a PISA value <130 mm^2^] (*n* = 16 for both groups) and group 2 (subjects with periodontal disease, i.e., a PISA value more than 130 mm^2^) (*n* = 11 for both groups). A PISA value of >130 mm^2^ has been shown to be associated with the Center for Disease Control and Prevention and the American Academy of Periodontology (CDC-AAP) case definition of periodontitis [23].

**Table 1:** Metadata of study subjects.

	**IgA ACPA high**	**IgA ACPA low**	***p*** **-value**
**Subjects (** ***n*** **)**	27	27	
**RA auto-antibodies in GCF**			
IgA ACPA (U/ml) (median, IQR)	0.40 (0.23–0.85)	0 (0–0)	**<0.0001** ^&^
IgG ACPA (U/ml) (median, IQR)	0 (0–0)	0 (0–0)	0.40^&^
IgA RF (IU/ml) (median, IQR)	0 (0–0)	0 (0–0)	0.51^&^
IgM RF (IU/ml) (median, IQR)	0 (0–0)	0 (0–0)	0.74^&^
**RA auto-antibodies in serum**			
IgA ACPA (U/ml) (median, IQR)	0.58 (0.37–0.82)	0.45 (0.35–0.80)	0.46^&^
IgG ACPA (U/ml) (median, IQR)	1.15 (0.86–2.26)	0.95 (0.60–1.61)	0.15^&^
IgA RF (IU/ml) (median, IQR)	1.96 (0.85–3.76)	2.00 (1.02–4.72)	0.97^&^
IgM RF (IU/ml) (median, IQR)	0 (0–0.69)	0 (0–0)	0.89^&^
**Demographic factors**			
Age in years, mean (SD)	43 (11)	44 (12)	0.76^$^
Female, *n* (%)	12 (44)	12 (44)	1
**Socioeconomic status**			
Education level (1–6)^*^ (mean, SD)	3.6 (1.6)	3.8 (1.5)	0.66^$^
**General health factors**			
**Co-morbidity:**			
Hypertension, *n* (%)	1 (3.7)	1 (3.7)	
Asthma		2 (7.4)	
**Medication:**			
None, *n* (%)	26 (96)	24 (89)	
Calciumchannelblocker, *n* (%)	1 (3.7)		
ACE inhibitor, *n* (%)		1 (3.7)	
Bronchodilator, *n* (%)		2 (7.4)	
BMI, mean (SD)	24 (4)	23 (3)	0.52^$^
**Lifestyle factors**			
Cigarette smoking, *n* (%)	9 (33)	9 (33)	1
Packyears (median, IQR)	20 (13–33)	20 (17–25)	0.83^&^
**Oral health factors**			
Number of teeth, mean (SD)	28 (5)	26 (5)	0.12^$^
PISA (mm^2^) (median, IQR)	39 (0–317)	8.4 (0–519)	0.67^&^
No periodontal disease^*^(group 1), ***n*** **(%)**	16 (59)	16 (59)	
PISA (mm^2^) (median, IQR)	9.5 (0–34)	0 (0–8.2)	0.18^&^
Bleeding on probing (% of sites) (median, IQR)	0.9 (0–3.3)	0 (0–1.1)	0.29^&^
Pocket probing depth ≥5 mm (% of sites) (median, IQR)	0 (0–1.9)	0 (0–1.1)	0.98^&^
Periodontal disease^*^(group 2), ***n*** **(%)**	11 (41)	11 (41)	
PISA (mm^2^) (median, IQR)	469 (234–1,034)	620 (317–1,113)	0.60^&^
Bleeding on probing (% of sites) (median, IQR)	31 (17–40)	33 (30–48)	0.35^&^
Pocket probing depth ≥ 5 mm (% of sites) (median, IQR)	9.1 (37–61)	6.5 (11–17)	0.29^&^

*Education level: 1. elementary school, 2. junior high school, 3. senior high school without diploma, 4. senior high school with diploma, 5. undergraduate, 6. postgraduate*.

* *According to cutoff of PISA > 130 mm^2^. A PISA value of >130 mm^2^ has been shown to be associated with the CDC-AAP case definition of periodontitis according to Leira et al. [23]*.

& *Mann–Whitney U-test*.

$ *Unpaired t-test with Welch's correction*.

### Downstream Analyses

**Figure 1:**
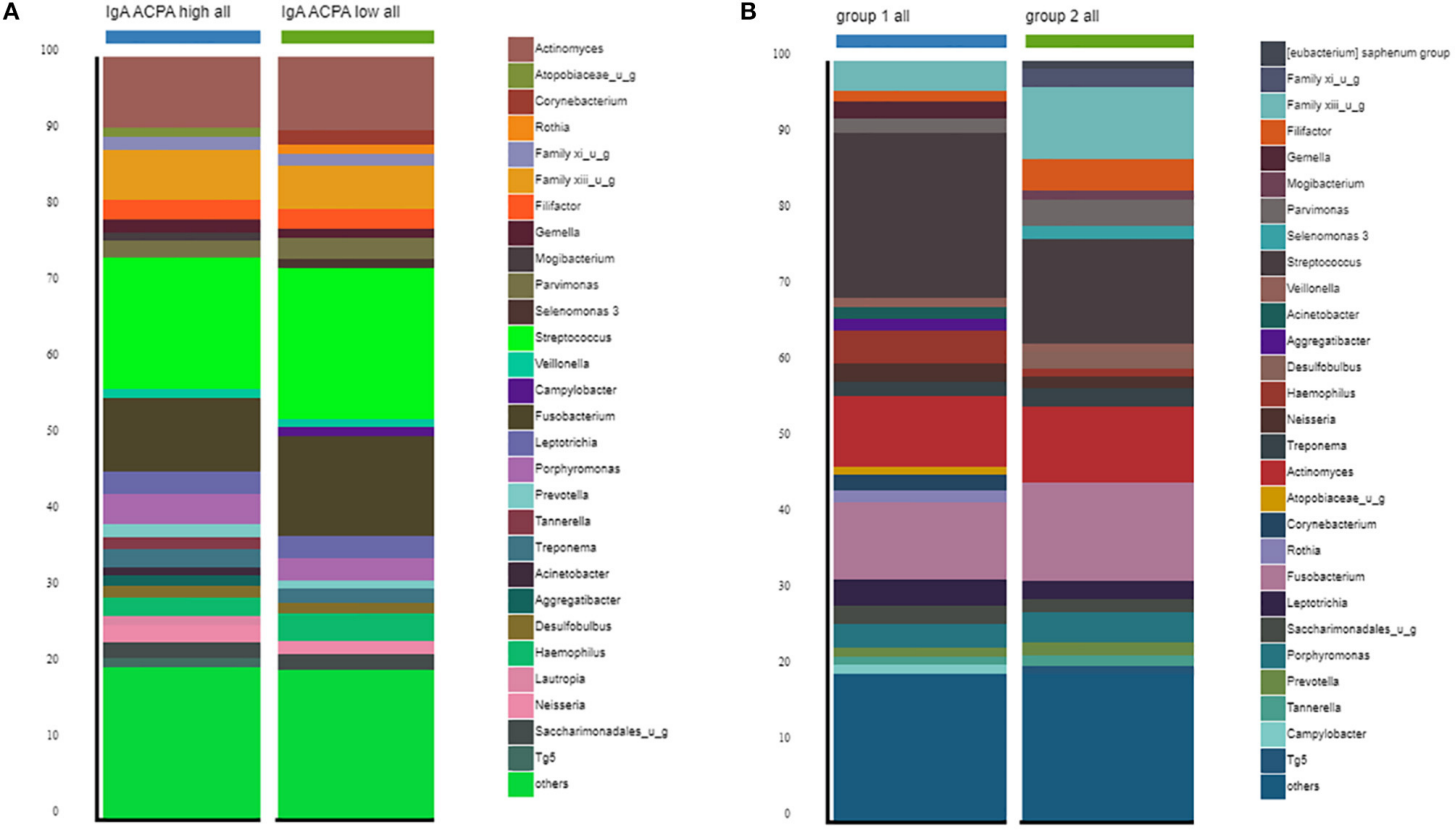
Top 30 features at genus level for subjects divided in **(A)** IgA ACPA high and IgA ACPA low and **(B)** subjects without periodontal disease (group 1) and subjects with periodontal disease (group 2).

Between the groups, no differences in alpha diversity were seen. The estimated median number of species per sample was around 170 (Table 2). Stacked bar figures show the top 25 features at genus level for IgA ACPA high and IgA ACPA low groups (Figure 1A) and between group 1 (without PD) and group 2 (with PD) (Figure 1B).

**Table 2:** Alpha and beta diversity between the groups.

**Group**	***n*** **(subjects)**	**Median nr. of reads (IQR)**	Median nr. of species^*^(IQR)	**Alpha diversity Shannon**	**Alpha diversity Chao1**
IgA ACPA high	27	92,840 (75,959–107,411)	174 (158–200)	0.216	0.158
IgA ACPA low	27	88,991 (75,193–106,986)	165 (148–186)		
Group 1 (HC without PD)	32	92,570 (74,923–105,246)	172 (150–189)	0.492	0.526
Group 2 (HC with PD)	22	85,919 (75,768–113,848)	172 (158–192)		
IgA ACPA high group 1	16	91,346 (69,418–101,857)	176 (152–19)	0.214	0.474
IgA ACPA low group 1	16	93,040 (79,518–106,946)	162 (150–182)		
IgA ACPA high group 2	11	104,841 (82,128–113,058)	172 (162–201)	0.768	0.212
IgA ACPA low group 2	11	79,016 (74,380–120,909)	167 (150–186)		

* *Chao1 index*.

**Figure 2:**
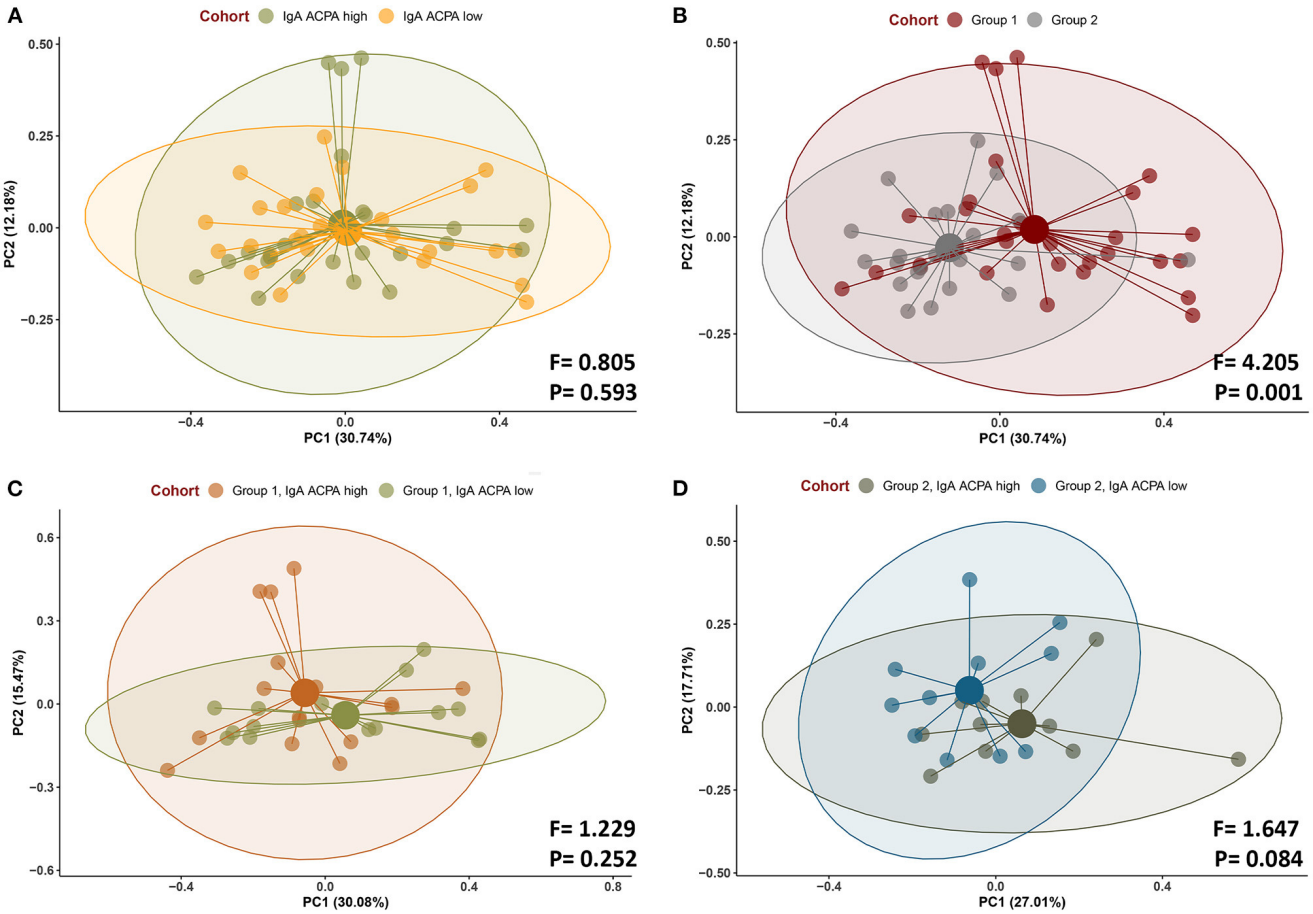
Beta diversity (Bray–Curtis) of subjects **(A)** with IgA ACPA high and IgA ACPA low, **(B)** without periodontal disease (group 1), and with periodontal disease (group 2) as well as **(C)** IgA ACPA high vs. low in subjects without periodontal disease (group 1) and **(D)** IgA ACPA high vs. low in subjects with periodontal disease (group 2, panel D).

Beta diversity was not significant when comparing IgA ACPA high vs. IgA ACPA low groups, but was significantly different between groups with or without PD (*F* = 4.205, *p* < 0.001) (Figures 2A,B, respectively). Beta diversity of subjects with PD (group 2) with high or low IgA APCA in GCF was borderline significant (*F* = 1.648, *p* = 0.084) (Figure 2D), while that was not the case in the group without PD (group 1) (Figure 2C).

### LEfSe Analysis

**Figure 3:**
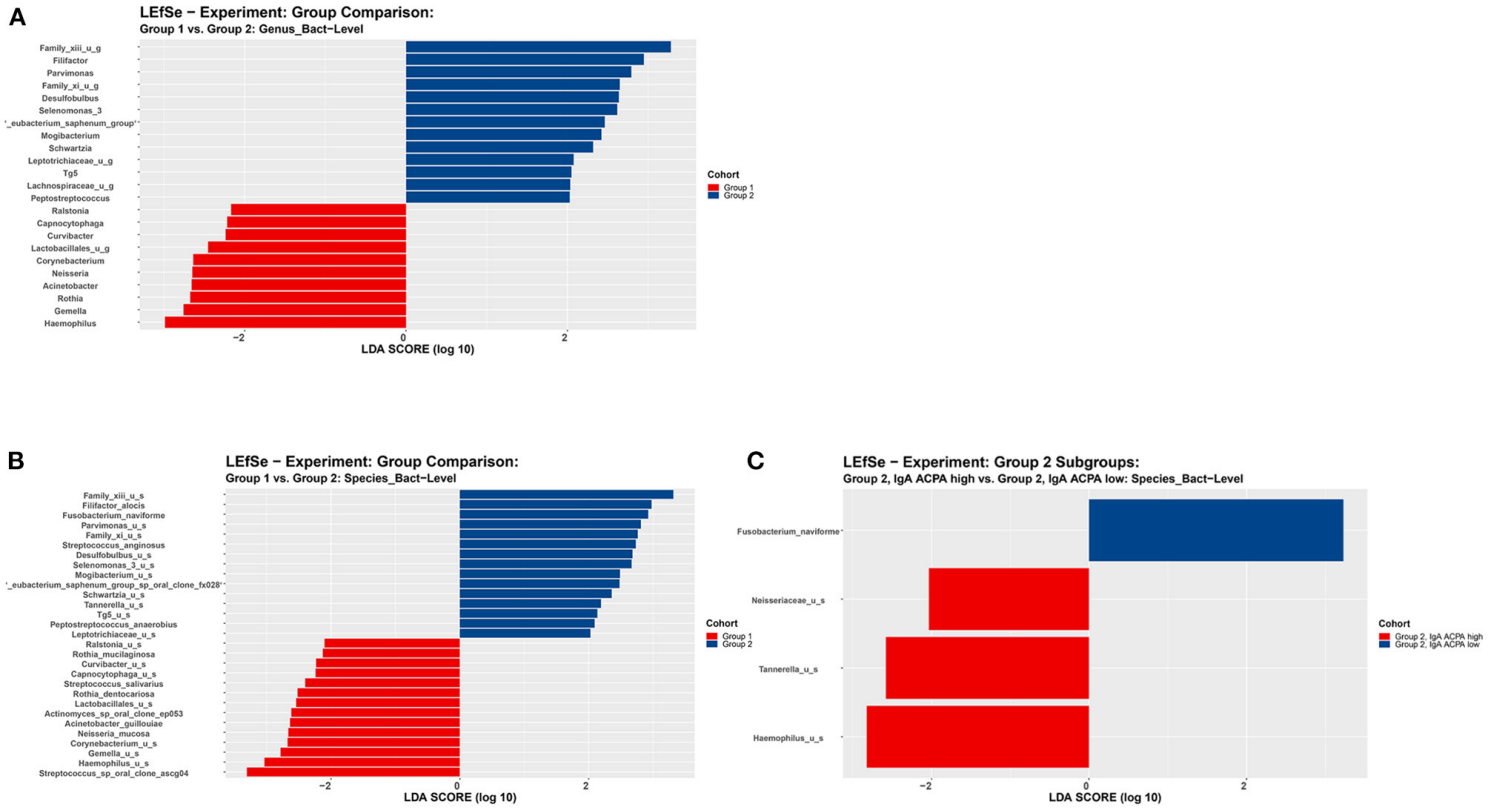
LEfSe analysis on **(A)** genus level and **(B)** species level of subjects without periodontal disease (group 1) and subjects with periodontal disease (group 2). **(C)** LEfSe analysis on species level of subjects with periodontal disease (group 2) with high or low IgA ACPA in GCF. All features shown meet *p* ≤ 0.05 for Kruskal–Wallis and Wilcoxon tests and have an LDA score ≥ 2.0 or ≤ −2.0. Red bars to the left convey that the feature in that group is more abundant in the “red” group than the other. Blue bars to the right convey that the organism is more abundant in the “blue” group.

Linear discriminant analysis effect size analysis on genus level revealed 13 features being more abundant in the subjects with PD compared to subjects without PD, including putative pathogenic taxa such as *Filifactor* and *Parvimonas* (Figures 3A,B).

No significant differences at genus level were found between subjects with high IgA ACPA compared to low IgA ACPA. However, zooming in on the subgroup with PD (group 2), LEfSe revealed the genera *Neisseriaceae, Tannerella*, and *Haemophilus* were more abundant in the IgA ACPA high group (Figure 3C).

### Features of Interest

**Figure 4:**
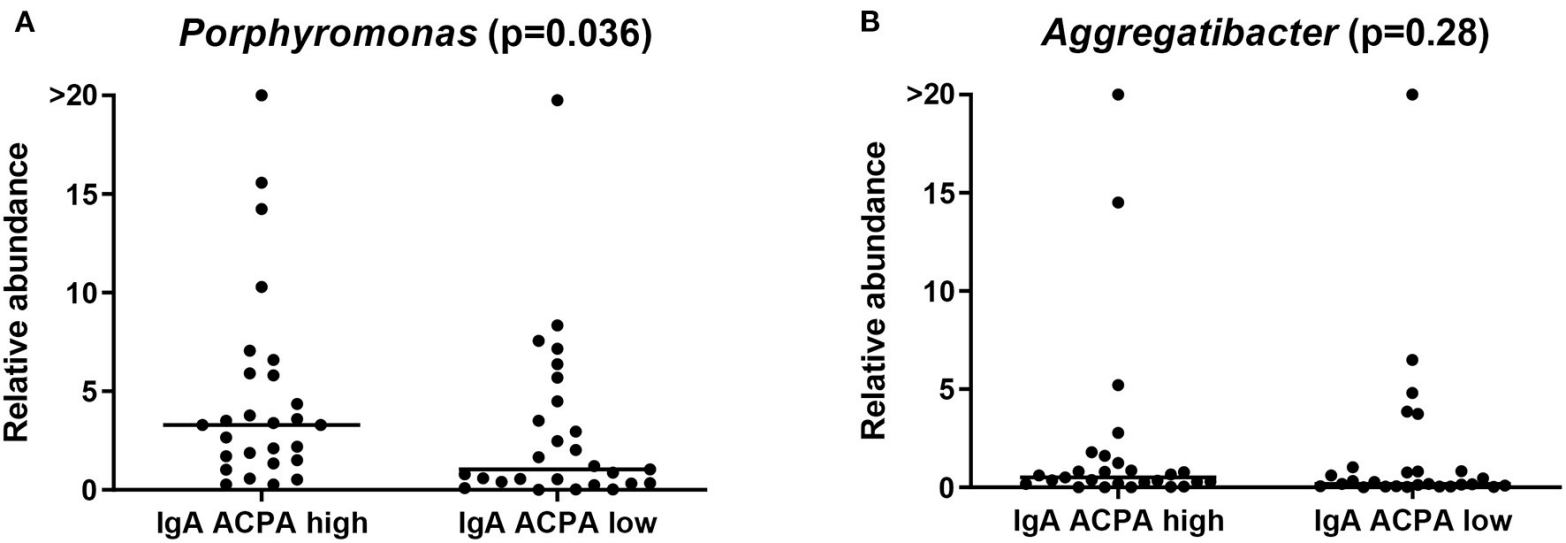
Relative abundance of the genus **(A)** *Porphyromonas* and **(B)** *Aggregatibacter* in healthy individuals with high or low IgA ACPA in GCF.

Although not identified by LEfSe, difference in the relative abundance of *Porphyromonas* and *Aggregatibacter* was additionally tested among the groups with a Kruskal–Wallis test. *Porphyromonas* was assessed at genus level (comprising *Porphyromonas* unidentified species, *P. endodontalis* and *P. gingivalis* ATCC 33277) to be as complete as possible. *Porphyromonas endodontalis* is indigenous to the oral cavity, and although PPAD was not identified in that species, some endogenous citrullination could not be excluded [24]. Relative abundance of *Porphyromonas* was significantly different between healthy individuals with high IgA ACPA in GCF compared to individuals without IgA ACPA in GCF (*p* = 0.036; Figure 4A).

The new genus *Aggregatibacter*, which was created to accommodate some former Haemophilus and Actinobacillus species (all members of the oral microbiota and implicated in oral and non-oral infection) [25], comprised *Aggregatibacter* unidentified species*, Aggregatibacter aphrophilus*, and *Aggregatibacter segnis*, and relative abundance of this genus was not different between the groups (Figure 4B). *Aggregatibacter actinomycetemcomitans* was not found at species level using the current pipeline.

## Discussion

The previous finding of increased levels of IgA ACPA in GCF of healthy individuals, which correlated with periodontal inflammation and smoking [17], led us to assess the possible relation with oral microbial dysbiosis. According to their periodontal status, the healthy individuals with or without IgA ACPA in GCF showed a significant difference in beta diversity, and LEfSe analysis revealed 13 pathogens at genus level to be enriched in subjects with PD, including *Filifactor* and *Parvimonas*. Comparison of these healthy subjects according to the presence of IgA ACPA in GCF did not show a significant differences in beta diversity, and LEfSe analyses revealed no enriched features in subjects with high IgA ACPA. No significant differences at genus level were found between subjects with high IgA ACPA compared to low IgA ACPA. Although not revealed by LEfSe, relative abundance of the genus *Porphyromonas* was higher in subjects with high IgA ACPA, whereas *Aggregatibacter* was not different. Taking a closer look at the subgroup with PD, despite the relatively small number of subjects (*n* = 22), there was a trend in beta diversity between the subjects with PD with or without IgA ACPA in GCF, and LEfSe analysis showed the genera *Neisseriaceae, Tannerella*, and *Haemophilus* to be enriched in subjects with high IgA ACPA. The found *Heamophilus* unidentified species could be related to the genus *Aggregatibacter* [25].

Periodontal pathogens with citrullinating properties have been described previously [11, 12], and also citrullinated proteins have been found in the periodontium [26]. Thus, the periodontium could be a site of production of antibodies to citrullinated proteins or ACPA. ACPA positivity is often seen as a prerequisite for the development of RA, and the local presence of ACPA has been described especially in mucosal tissues [16]. In 2012, Scher et al. were one of the first to investigate periodontal disease and oral microbiota in patients with new-onset RA compared to patients with established RA and healthy controls [27]. They found that the subgingival microbiota of patients with new-onset RA were similar to that in patients with RA and healthy controls, whose PD was of comparable severity, and that the presence of *Porphyromonas gingivalis* was not different between patients with RA and controls. Since then, many studies have been published comparing microbiota in patients with RA and osteoarthritis (OA) [28, 29], patients with RA and healthy controls [30], or in patients with RA only [31, 32]. Mikuls et al. could not identify a subgingival microbial fingerprint that could reliably discriminate RA from patients with OA, whereas Chen et al. found 8 oral bacterial biomarkers that could differentiate RA from OA (*Actinomyces, Neisseria, Neisseria subflava, Haemophilus parainfluenzae, Haemophilus, Veillonella dispar, Prevotella, and Veillonella*) [28, 29]. Corrêa et al. reported that patients with RA without PD had enrichment in periodontitis-associated bacteria such as *Prevotella*, whereas pathogenic taxa such as *Prevotella, A. actinomycetemcomitans*, and *Parvimonas micra* were also significantly increased in patients with RA with PD compared to control subjects with PD only [30]. Eriksson et al. found many differences in microbial profile between patients with RA with or without PD and also the differences between bacterial species in plaque compared to saliva [32]. Also, Beyer et al. saw different microbiota compositions associated with different levels of gingival bleeding, periodontal probing depth, RA disease status, prednisolone use, and smoking [31]. Lopez-Oliva et al. compared microbiota in periodontally healthy patients with RA and non-RA [33]. As expected from a periodontally healthy cohort, *Porphyromonas gingivalis* and *A. actinomycetemcomitans* were not significantly different between groups; however, *Cryptobacterium curtum*, an organism capable of producing L-citrulline, although as a single amino-acid and not likely to be engaged by the major histocompatibility complex, emerged as a robust discriminant of the microbiota in individuals with RA [33]. In all these studies, often, different species of interest were found, and also, relation of *Porphyromonas gingivalis* or *A. actinomycetemcomitans* to RA was not uniformly confirmed.

More recently, studies have been focusing on early onset RA or patients at risk for RA, the latter usually defined by the presence of ACPA in serum and arthralgia. Early patients with RA with symptoms <12 months showed independently of periodontal status enriched levels of *Prevotella pleuritidis, Treponema denticola, Porphyromonas endodontalis*, and *Filifactor alocis* species compared to healthy controls [34]. Cheng et al. compared early RA and at risk patients with RA to healthy controls and showed dysbiosis, including an increase of *Porphyromonas gingivalis*, in the periodontally healthy microbiota (and altered diseased subgingival microbiota) of at risk RA compared with HC [35]. Kroese et al. reported similarities in oral microbiota of early patients with RA and patients with RA at risk, since in both groups, the oral microbiota were characterized by an increased relative abundance of potentially proinflammatory species when compared to that in healthy controls [15]. *Prevotella salivae, Veillonella*, and *Prevotella* were more abundant in the early RA group and RA at risk group compared to the healthy control group. The genus *P gingivalis* was not identified in this study as a discriminative microorganism [15]. The differences in outcomes between the various studies can be explained by differences between study groups, such as including patients at different stages of RA, differences in disease activity, differences in therapy, and including patients at risk or early and established RA. Also, the site of sampling of microbial specimens differs between studies, such as saliva, subgingival plaque, or GCF. This makes it difficult to compare the outcomes of all these studies.

Previously, antibodies to periodontal pathogens have been investigated and related to RA autoantibodies, but often weak, or no correlations of anti- *Porphyromonas gingivalis* antibodies with ACPA or RF were found [20, 36]. In a large population of non-RA persons of 60 years and older tested for antibodies against 19 periodontal species, no association with RF seropositivity was found [37]. Our group investigated anti-*Porphyromonas gingivalis* antibodies in seropositive arthralgia patients and found that these antibodies did not predict development to RA [38].

To our knowledge, this study is the first to compare local_presence of IgA ACPA in the periodontium to the subgingival microbiota in healthy subjects with or without PD. According to the mucosal origins hypothesis [16], these subjects, specifically those with PD, could be at risk for developing RA. Studies comparing oral microbiota to systemic ACPA levels have been published. Tong et al. investigated oral microbiota in saliva samples of ACPA seropositive individuals at risk for developing RA and found that serum ACPA concentration was positively correlated with the relative abundance of *Eubacterium nodatum*_*group, Peptostreptococcus, Tannerella, and norank_o__Absconditabacteriales_SR1*, while conversely associated with *Haemophilus Neisseria* [39]. Unexpectedly, the relative abundance of *Porphyromonas gingivalis* was significantly decreased in the RA at risk individuals. Kroese et al. compared oral microbiota of ACPA seropositive vs. ACPA seronegative RA at-risk individuals and could not find any differences [15].

## Conclusion

In a cohort of healthy subjects with or without IgA ACPA in GCF, significant compositional differences in the subgingival microbiota were seen according their periodontal status. Comparison between presence or absence of IgA ACPA in GCF revealed no compositional differences, although relative abundance of *Porphyromonas* was higher when IgA ACPA was present. Of note, within the subjects with PD, the presence of IgA ACPA in GCF was associated with *Neisseriaceae, Tannerella*, and *Haemophilus*. This implicates that PD and certain taxa seem to be associated with local presence of ACPA in the periodontium, supporting the mucosal origins hypothesis for development of RA.

## Data Availability Statement

The datasets presented in this study can be found in online repositories. The names of the repository/repositories and accession number(s) can be found below: https://figshare.com/articles/dataset/IgA_ACPA_in_GCF/19640868.

## Ethics Statement

The studies involving human participants were reviewed and approved by the Medical and Health Research Ethics Committee of the Medical Faculty of Gadjah Mada University, Yogyakarta, Indonesia, according to the Declaration of Helsinki 2008 (Ref: KE/FK/430/EC, April 29, 2014). The patients/participants provided their written informed consent to participate in this study.

## Author Contributions

MJdS, AV, and JW: conceptualization and project administration. MJdS and JW: methodology, writing—original draft preparation, writing—review and editing, and supervision. AV, JW, and HJMH: validation, data curation, and funding acquisition. MJdS, PSR, ER-E, and PL: formal analysis. PSR, ER-E, HJMH, and PL: investigation. AV: resources. MJdS, HJMH, NK, AV, and JW: visualization. All authors have read and agreed to the published version of the manuscript.

## Funding

This study was funded by the author's institutions, and partly by the Dutch Arthritis Foundation (ReumaNederland, Grant Number 16-2-201), as well as an Abel Tasman Talent Program Sandwich Ph.D. Grant from the Graduate School of Medical Sciences of the University of Groningen.

## Conflict of Interest

The authors declare that the research was conducted in the absence of any commercial or financial relationships that could be construed as a potential conflict of interest.

## Publisher's Note

All claims expressed in this article are solely those of the authors and do not necessarily represent those of their affiliated organizations, or those of the publisher, the editors and the reviewers. Any product that may be evaluated in this article, or claim that may be made by its manufacturer, is not guaranteed or endorsed by the publisher.
